# Ultrasound contrast agent assisted ultrasonography guidance percutaneous nephrostomy for non-hydronephrotic kidney

**DOI:** 10.1186/s13089-024-00362-9

**Published:** 2024-02-22

**Authors:** Weijie Jiao, Xue Gong, Yuanyuan Sun, Lin Sang, Xiaoying Ding, Ming Yu

**Affiliations:** The Department of Ultrasound, Xijing Hospital, The Fourth Military Medical University, No.127 Changle West Rd, Xi’an, 710032 Shaanxi China

**Keywords:** Ultrasonography (US) guidance, Percutaneous nephrostomy (PCN), Ultrasound contrast agent (UCA), Non-hydronephrotic kidney

## Abstract

**Background:**

Given the limited success rate and considerable challenges associated with conventional ultrasonography (US) guidance for percutaneous nephrostomy (PCN) in non-hydronephrotic kidneys, this study proposed a solution with ultrasound contrast agent to enhance the success rate and mitigate the difficulties.

**Materials and Methods:**

From January 2017 to August 2023, a total of thirteen patients diagnosed with non-hydronephrotic kidney were included in the study. Following routine ultrasonography examination, no significant dilatation of the renal collecting system was observed. US-guided percutaneous nephrostomy PCN was performed with the assistance of ultrasound contrast agent (UCA). The patients were subsequently monitored to assess the improvement of symptoms and postoperative recovery.

**Results:**

The success rate was found to be 100% for all patients (13/13) and kidneys (20/20). The average volume of UCA solution used was 19 ± 6.7 mL (range, 11–35 mL), while the mean duration of the operation was 18.92 ± 8.96 min (range, 7–36 min). A majority of the patients (12/13) underwent a single puncture procedure. Throughout the follow-up period, no serious complications were observed, and surgery resulted in significant alleviation of symptoms in all patients.

**Conclusion:**

The use of UCA-assisted US guidance PCN has been shown to be effective in achieving urinary diversion and alleviating associated clinical symptoms in non-hydronephrotic kidneys. In comparison to traditional methods, this approach demonstrates a high success rate and safety profile, while also offering a simplified operative procedure. Consequently, it presents a novel method and concept for managing non-hydronephrotic kidneys afflicted by urine leakage.

## Introduction

The initial ultrasonography (US) guidance percutaneous nephrostomy (PCN) was carried out by J.F. Pedersen, which pioneered another guidance for PCN after fluoroscopy and is equally famous with computed tomography (CT) [1–3]. Nowadays, US is considered as a dependable guidance approach for PCN due to its lower cost, nonionizing radiation and real-time, excellent, cross-sectional anatomic details etcetera [4, 5]. US guided PCN can be successfully performed in 96.6–100% of kidneys with dilated renal collecting system and with low complication ratio [5–8]. Nevertheless, when it comes to kidneys with nondilated collecting system, the technical success ratio distinctly decreases to about 80% [7]. Meanwhile, compared with patients with hydronephrosis, it is reported that the overall complication ratio can increase sixfold in performing PCN among non-hydronephrotic patients [9].

In recent years, with the continuous application of abdominopelvic surgery, the incidence of iatrogenic damnification (e.g., ureter or bladder injury) is soaring [10–12], causing urine leakage occurred or urine flow into the abdominal cavity. Besides, the occurrence of urine leakage can be attributed to radiation damage (e.g., radiocystitis) or traumatism (e.g., pelvis fracture). Furthermore, lower urinary tract obstruction may arise in patients with acute kidney injury resulting from prostate surgery. Consequently, patients' kidneys exhibit either no or minimal hydronephrosis. Thus, it is imperative to promptly identify a suitable and viable approach to implement percutaneous nephrostomy (PCN) in non-hydronephrotic kidney patients.

The preparation and modification of ultrasound contrast agent (UCA) has become a cynosure topic in the field of ultrasonic medicine in recent years [13]. Among them, sulfur hexafluoride micro bubbles (MBs) has an excellent persistence time in the vasculature application due to several advantages, including high core molecular weight gasses and do not diffuse across the protein shell [14]. Since sulfur hexafluoride MBs can enhance the cavitation effect and thus promote ultrasonic biological effects, it is widely utilized in ultrasonic thrombolysis [15], high-intensity focused ultrasound [16] and have a promising future in US intracavitary diagnose [17, 18].

The objective of this study is to propose a unique approach for patients with non-hydronephrotic kidney to achieve urinary diversion and alleviate clinical symptoms. Briefly, US was utilized to initially investigate the trajectory and puncture site for PCN. Subsequently, UCA was injected into the renal pelvis to aid in imaging and confirm the accurate placement of the puncture needle within the target renal calyx. Finally, catheter dilation was performed to facilitate urine drainage and evaluate any potential complications.

## Materials and methods

### Participated patients

From January 2017 to September 2023, 688 patients in our hospital need to underwent PCN were preliminary selected. Scrutiny and record the basic information of all patients (gender, age, clinical symptoms, etc.). Criteria applied for patients’ inclusion to carry out UCA assisted US guided PCN group was shown in Table 1 and the basic information of the selected study sample was manifested in Table 2, respectively.

**Table 1:** Criteria for inclusion to performed UCA assisted US guided PCN group

Clinical necessity to urinate, urethral drainage, urinary diversion or relieve various clinical symptoms caused by urine leakage, etc
No significant dilatation of the renal collecting system^a^ was confirmed in all patients after repeated routine US examination
No severe disorders of blood coagulation, cardiopulmonary insufficiency and pulmonary function

^a^Grade 0 or 1 renal appearance on the four-grade system used to classify the degree of hydronephrosis with maximum 2–3 mm calyceal separation [19, 20]

**Table 2:** Basic information of the study population

Characters of sample	Value
Mean age, year	58.46 ± 17.35 (37–88 years)
Gender	
Male	5
Female	8
Clinical symptom	
Urine leakage	9
Seroperitoneum	1
Acute peritonitis	2
Electrolyte disturbances accompanied with hypourocrinia	1
Cause of symptom^a^	
Traumatism	1 (2)
Iatrogenic bladder fistula	4 (5)
Radiational damage	1 (2)
Acute kidney injury	1 (2)
Iatrogenic ureteral fistula	6 (9)

^a^The number of kidney due to the corresponding cause in parentheses

**Fig. 1:**
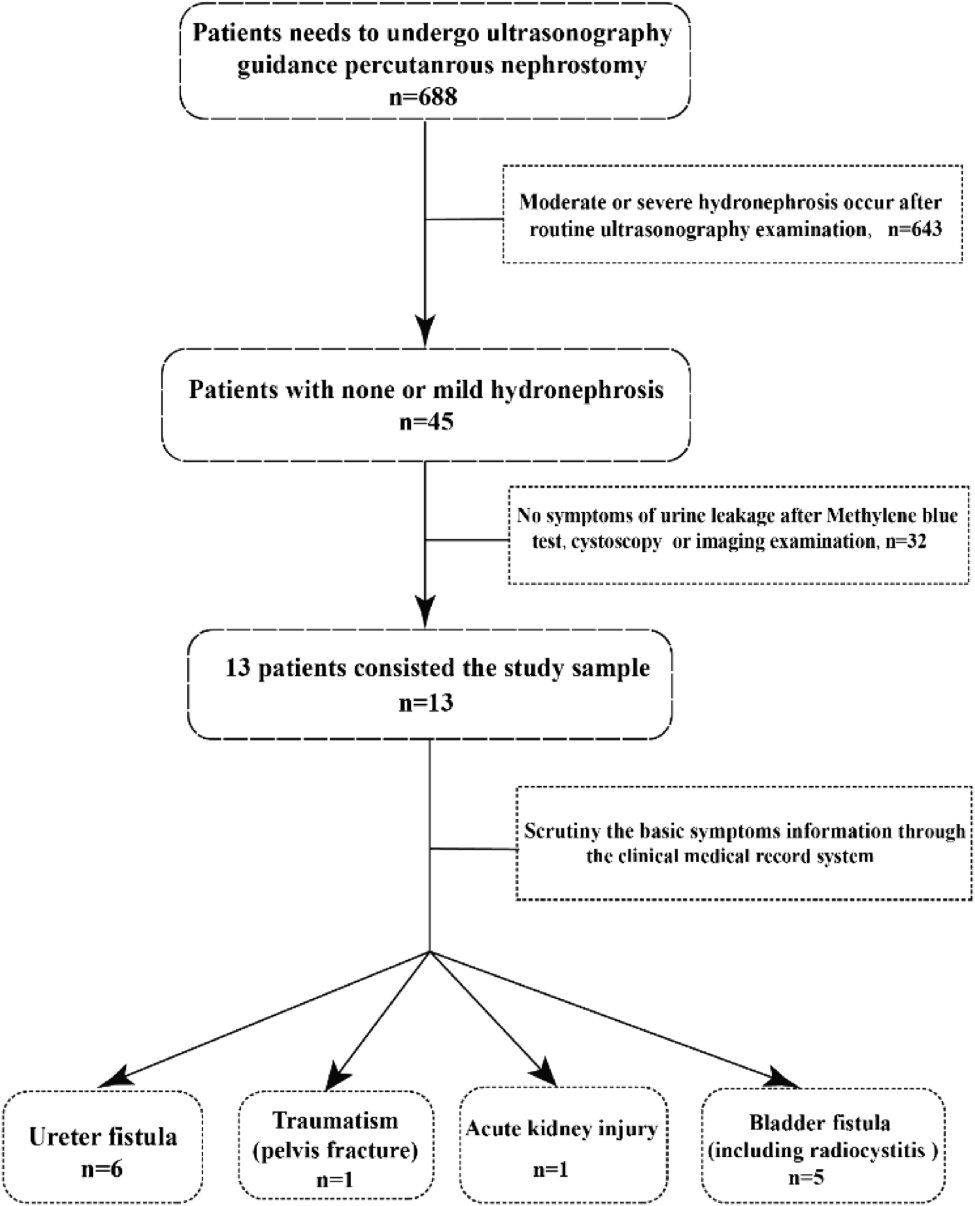
The flowchart of patient screening procedure

Figure 1 delineates the screening procedure, whereby a total of 13 participants with non-hydronephrosis were included in the study sample. This sample comprised 5 male and 8 female individuals, with a mean age of 58.46 ± 17.35 years (range, 37–88 years). Furthermore, the study sample encompassed a total of 20 kidneys.

### Ultrasonography apparatus and probe

Ultrasound images were obtained utilizing the Philips EPIQ 7 system. Linear array probes and convex array probes were employed for ultrasonic examination. Specifically, the linear array probe operated at frequencies of 3–12 MHz and 5–18 MHz, while the convex array probe operated at a frequency range of 1–5 MHz.

### Technique of UCA assisted US guided PCN

All procedures were performed by two urologists with more than 10 years of experience in performing PCN. Meanwhile, two sonographers with more than 7 years of experience in performing UCA assisted with administration of US guidance intervention.

*Step 1 Perpetration of UCA solution*—The UCA applied in this study is sulfur hexafluoride MBs (SonoVue, Bracco Suisse SA, Switzerland). Briefly, 5 mL of 0.9% sodium chloride solution was injected into a bottle of sulfur hexafluoride MBs and shake it slightly to make a dispersion solution [21]. Next, a 1 mL sample of the thoroughly agitated sulfur hexafluoride MB solution was extracted and subsequently injected into 100 mL of normal saline in order to produce a diluted UCA solution for future application.

**Fig. 2:**
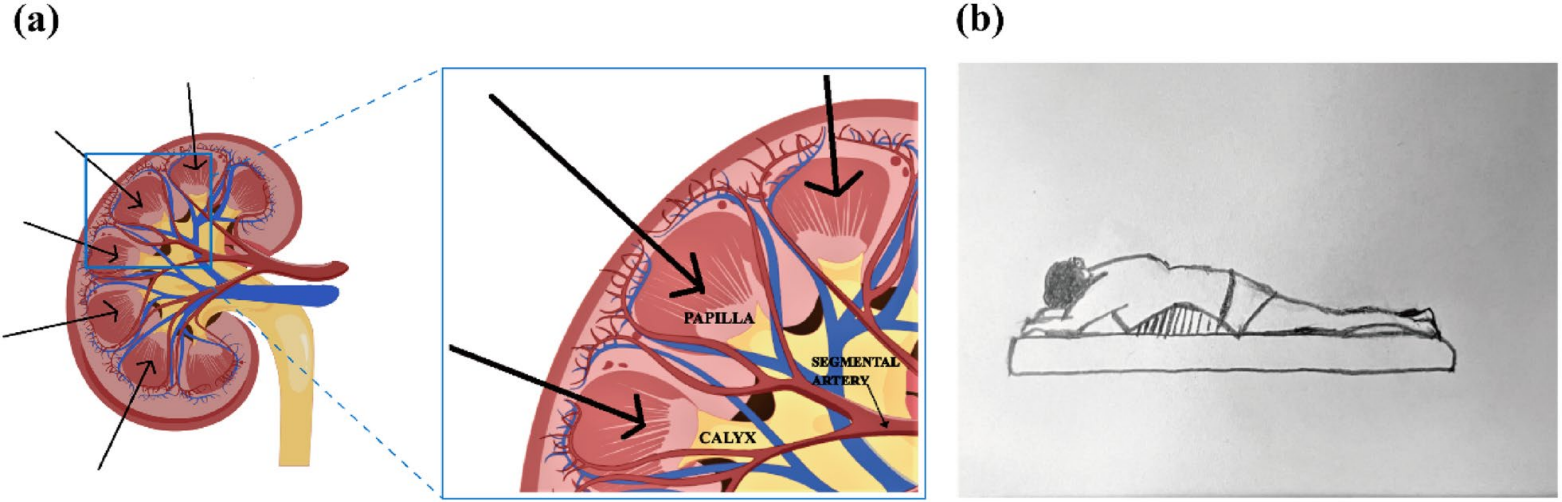
Diagram of puncture needle insertion course and patients’ potion. **a** the insertion course (black arrow) should avoid the blood vessels of renal calyx. The schemes were powered by Figdraw; **b** Patients lies prone in the prone position. This diagram was draw by author

*Step 2 Puncture needle insertion course and patients’ position*—Based on the renal anatomical structure and vascular distribution, it is recommended that the needle be inserted perpendicularly at the puncture site, traversing the layers of skin, subcutaneous tissue, renal capsule, renal pyramid, and renal medulla. This approach aims to minimize the distance of needle insertion and mitigate potential harm, as Fig. 2a shown. Prior to needle insertion, it is imperative to conduct a Color Doppler ultrasound examination to ascertain the absence of any significant blood vessels at the intended site of insertion.

*Step 3 Puncture*—Patients were placed into prone position, as Fig. 2b shown. Injected 5 mL of 1% Lidocaine Hydrochloride solution (Harvest Pharmaceutical, CO., LTD, Shanghai, China) for infiltration anesthesia. Cut skin with a No.11 sharp knife to the level of half the blade. Under the guidance of US, the 18-gauge × 15 cm PTC needle (Hakko Sonoguide, Japan) was placed into the puncture guide groove. It is of great necessity to exhort patients to hold their breath and slowly insert the needle along the direction of the puncture frame. When the image displays the precise location where the puncture needle penetrates the tip of the renal pyramid, the needle core should be withdrawn. Subsequently, the prepared UCA media should be injected into the patients. By injecting the diluted UCA solution, the structures of the renal calyx and renal pelvis can be clearly visualized in the resulting images.

*Step 4 Dilating catheter *—A 0.035 inch × 80 cm guidewire (Radifocus, Terumo Corporation, Tokyo, Japan) was insert into the renal pelvis through the PTC needle sheath. Withdraw the needle sheath, inserting the arterial sheath (Radifocus Introducer II, Terumo Corporation, Tokyo, Japan) along the guidewire direction for dilation. Retracted the inner core and withdraw the urine could suggested that the dilating catheter progress was accomplished. Then, retracted the sheath, place the BARD drainage catheter (Bard Access System, USA) into the renal pelvis along the guidewire. Remove the guidewire and the fixed soft core of the drainage catheter gently when the part of the drainage catheter enters the kidney. Tighten the tip of the catheter to secure the wire and close the latch, fixing the drainage catheter and stick a sterile dressing to the wound at the puncture point and connecting Discofix (Braun Melungeon, Germany) and anti-reflux drainage pack.

### Assessment of complications

All patients were prospectively monitored to document the 24 h urine drainage volume of the fistulectomy catheter, the incidence of complications, and the amelioration of clinical symptoms, among other variables. Patients were contacted daily throughout their hospital stay. Upon discharge, follow-up was limited to subsequent visits for medical attention.

### Data collection

Record the significant images of the operation process. The success ratio is measured through whether the drainage catheter is successfully placed into the destination renal calyx and whether the urine can be successfully extracted or not. Meanwhile, the total dose of used UCA solution for each patient was recorded to calculate the mean value and the complete operation time were noted. All data were analyzed through GraphPad Prism software (version 8.0.2, San Diego, United States). Mean ± standard deviation was utilized to describe quantitative variables.

## Results

### Success ratio

**Fig. 3:**
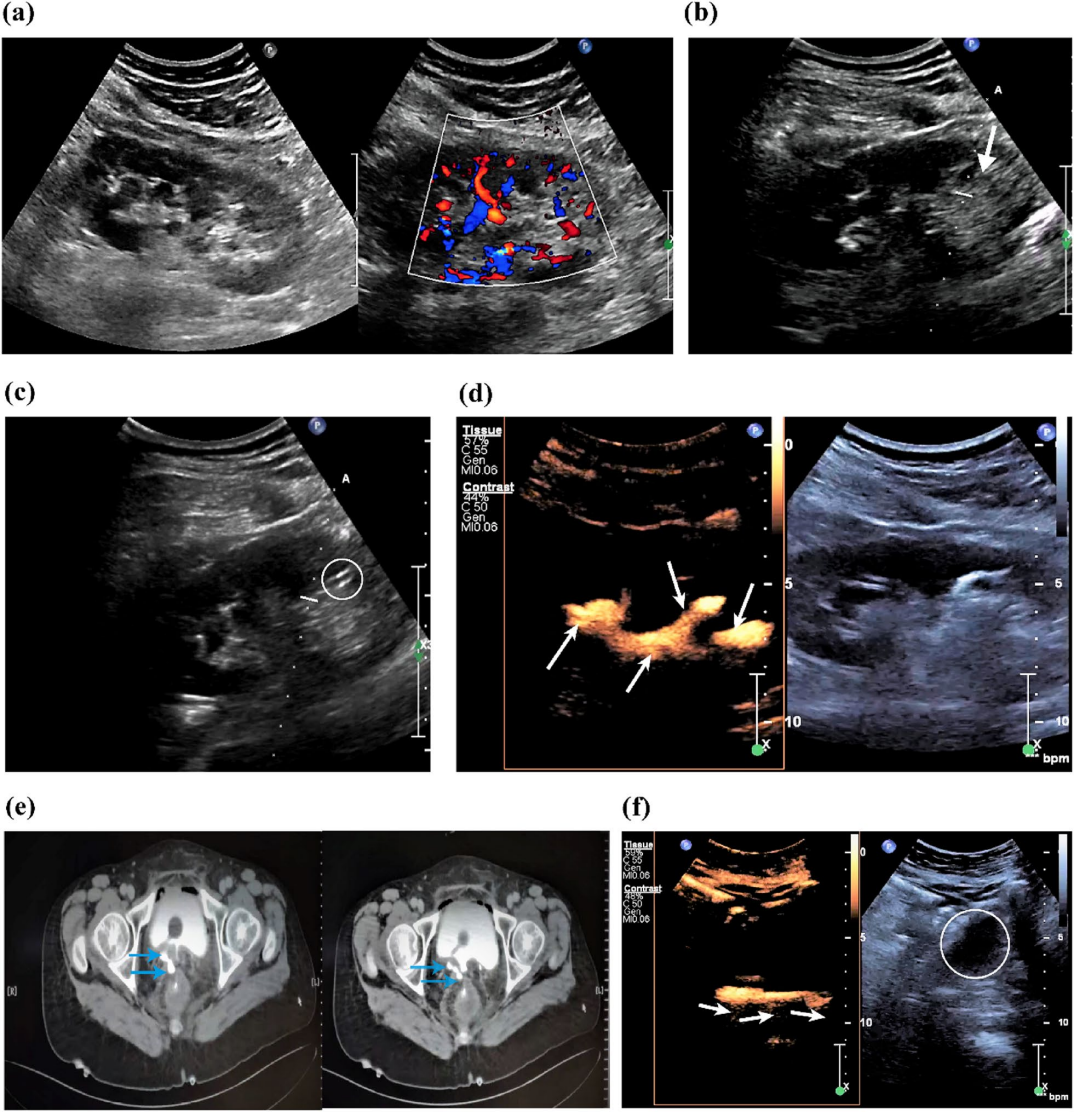
Images of a 53 year-old woman, diagnosed with ureteral fistula, underwent UCA assisted US guided PCN. **a** Conventional US and Doppler US demonstrate that none hydronephrosis occurs in renal system. **b** The course (arrow) of PTC puncture needle insertion. **c** The needle tip has entered the destination renal papilla (circle). **d** Withdraw the PTC needle, the renal calyces and its pelvis structures can be clearly observed via the diluted UCA solution be injected (arrowheads). **e** CT results manifested that the right ureter near the bladder opening was unclear and a little contrast medium was visible in the pelvis (arrowheads). **f** UCA media only presents in the patient's ureter (arrow) and not in the bladder (circle), suggesting ureteral fistula occurred

With the assistance of sulfur hexafluoride MBs, all US guided PCN were triumphantly carrier out on each patient (13/13) and each kidney (20/20). The success ratio was 100%. The mean duration of operation procedure, the mean usage of the UCA solution was 18.92 ± 8.96 min (range, 7–36 min) and 19 ± 6.7 mL (range, 11–35 mL) respectively. Moreover, the average number of punctures for per kidney is 1.08 ± 0.28 (range, 1–2 frequency). Nineteen kidneys (95%, 19/20) belonging to twelve patients (92.31%, 12/13) were effectively punctured during the initial insertion. The sole exception was an elderly male patient aged 68, who experienced two unsuccessful punctures in the right kidney. Figure 3 specifies the operation procedure of a patient (female, 53 years old) diagnosed with ureteral fistula.

### The volume of urine drainage

The average 24 h urine drainage volume of patients is 1446 ± 239.3 mL (range, 1100–1800 mL), suggesting the urinary function of patient's kidneys is normal and urine can be successfully drain through the catheter. A subset of patients (3 out of 13, 23.1%) experienced pale hematuria following the insertion of the drainage catheter. However, the urine color of these patients returned to its normal pale yellow or limpid state within 24 h. The average duration of recovery was found to be 11.31 ± 5.39 h (with a range of 6 to 24 h). Conversely, the remaining nine patients (10 out of 13, 76.9%) exhibited clear or yellow urine. Over the course of follow-up, the volume of urine drained through the fistulectomy catheter increased in all patients, reaching its peak between days 5 and 7.

### Date information of follow-up period

The average days of follow-up period was 12.31 ± 7.1 (range, 4–25 days). Postoperative symptom improvement was assessed through conventional US examination during follow-up period. The detail improvement of clinical symptom is shown in Table 3**.** During follow-up period, none serious complications, such as sepsis, septic shock, necrosis etcetera, occurred among all patients. The examination results manifested that the clinical symptoms for the majority patients (10/13, 76.92%) were improved within a week and the leakage of urine was greatly reduced until it disappeared.

**Table 3:** Clinical symptom improvement detail

Clinical symptom	Improvement status
Urine leakage	Urine leakage scenario decreases gradually until it disappears
Seroperitoneum	Seroperitoneum decreases gradually until disappears
Acute peritonitis	Inflammation symptom alleviated; infection index drops until return to normal
Acute kidney injury	Renal function recovers, creatinine decreases and urine volume increases

Only three patients (3/13, 23.08%) occurred minor complications. One patient, suffered from radiocystitis, accompanied with bladder fistula, incomplete ileus and acute peritonitis, underwent a longer postoperative recovery period (25 days). This patient occurred hematuria lasted 24 h and the urine color returned to normal on day 3. After 22 days’ conservative treatment, this patient was discharged. The nephrostomy catheter of a single patient experienced a minor blood leakage because of dual puncture. Administration of 2U Hemocoagulase Bothrops Atrox via intramuscular injection successfully ceased the blood leakage from the catheter. Another patient, who was of advanced age, developed a perirenal hematoma. The patient was monitored for a duration of 5 days, during which US images indicated a gradual reduction in the size of the hematoma until complete resolution.

## Discussion

Iatrogenic injury is the most common cause of damage to the ureters, accounting for about 75% of all ureteric injuries [22]. Early recognition and repair of injury during primary surgery can result in reduced morbidity, increased ease of repair, and a more favorable outcome for the patient. However, only 33% ureteric injuries are diagnosed during the primary procedure [22]. An unrecognized ureteric injury can lead to serious complications, including urinoma, abscess, ureteric stricture, ureteric fistula and potential loss of an ipsilateral renal unit [10]. In the majority of cases involving ureter or bladder injuries, particularly among female patients, urine does not exit through the urethra but instead collects in adjacent areas such as the perineum, vagina, or rectum. To mitigate their distress and enhance their overall well-being, it is imperative to redirect the flow of urine.

Nowadays, US-guided puncture and fluoroscopy or CT guidance subsequently during drain insertion are widely considered as the gold standard for nephrostomy [4, 23, 24]. However, the utilization of fluoroscopy or CT guidance in certain procedures may present limitations, such as complex operation procedures and limited availability at the bedside. Additionally, while the radiation exposure associated with these guidance techniques is minimal, it is essential to adhere to the principle of minimizing radiation exposure, known as the ‘‘as low as reasonably achievable’’ principle. In contrast, ultrasound US holds significant significance in percutaneous nephrostomy PCN procedures. It is effective in (i) assessing the degree of hydronephrosis, (ii) locating the most appropriate calyx for puncture and (iii) revealing the presence of stones or blood clots or other lesions [25]. However, as Patel et al. reported, a nondilated renal collecting system presents two difficulties: poor visualization and absence of distention [19]. Meanwhile, the success rate of primary PCN has been reported to be 98.2% in dilated renal systems, but only 82% in nondilated renal systems [26].

As one of the most common utilized UCA, sulfur hexafluoride MBs were widely applied in a variety of clinical research for the intracavitary use and the safety is high. In 2012, Zhou et al. and Xu et al. took the lead in applying sulfur hexafluoride MBs to the imaging of intrahepatic bile ducts [21, 27]. Besides, Ignee et al. applied UCA for intracavitary use to percutaneous transhepatic cholangiography and drainage in 38 [24]. Moreover, intra-cavitary use of UCA is considered safe and accurate in contrast-enhanced voiding urosonography [28, 29], percutaneous nephrolithotomy [30], respectively. Cui et al. and Jung et al. have also reported the application of UCA in US guiding PCN, particularly in non-collecting system dilated renal PCN [25, 31].According to their conclusion, intracavitary US with contrast enhancement shows promise as a novel technique for guiding percutaneous nephrostomy (PCN) and evaluating complications associated with catheter insertion. In fact, these research backgrounds is exactly where our inspiration came from.

The most challenging issue encountered during the puncturing procedure of a non-hydronephrotic kidney pertains to accurately determining the entry of the PTC needle into the renal calices. Unlike in hydronephrotic kidneys, the position of the needle tip is not easily observable. However, this lack of visibility is advantageous in the context of the essential need to puncture the renal cavity and administer the UCA solution. If the media is quickly distributed in the punctured calyces and both calyces and renal pelvis are observed under ultrasound guidance, it can be determined that the needle tip is in the correct position. Conversely, if the UCA solution is detected in the renal parenchyma, it indicates that the needle is not properly positioned, and adjustment of the needle tip is necessary. Furthermore, UCA offers an advantage over iodinated contrast material by effectively eliminating microbubbles in cases of inaccurate placement. As a result, it prevents the formation of a disruptive mass of contrast material that could impede the procedure.

The number of punctures and operation duration required for PCN primarily depend on the specific guiding instrument applied and the degree of renal hydrops. In general, there is a positive correlation between the total operation time and the number of punctures. Egilmez et al. reported that more punctures are needed for patients without hydronephrosis [32]. The occurrence of three CT-guided punctures differed among patients with grades 0–1, grade 2, and grade 3 hydronephrosis, with prevalence rates of 68%, 10%, and 0%, respectively. When transitioning from grade 3 to grades 0–1, there was a notable escalation in the median duration from 14 to 20 min. Sommer et al. needed a mean number of 3.6 ± 2.6 using combined CT and fluoroscopy to guide PCN in patients with nonobstructive uropathy due to urine leaks in cases of failed US-guided procedures [33]. The duration of the complete procedure was lasted as long as 87 ± 32 min. Still, when PCN was guided by US for patients with dilated collecting system, the average length of the procedure was only 9.07 ± 2.79 (range, 6–15 min) and only 9.66% of patients required more than a single attempt of puncture [6]. Compared with these studies, the average number of punctures and operation time in this study are attractive, which is 1.08 ± 0.28 frequency and 18.92 ± 8.96 min, respectively. Only one older patient who had suffered twice punctures in the right kidney and the duration lasted for 36 min.

There are still some limitations in our research. Initially, one major drawback of this research lies in the absence of a control group receiving conventional US guidance to assess the potential value of contrast usage. Besides, certain findings only consist of descriptive data and lack a standardized measure of accuracy, such as the evaluation of complications. Nevertheless, the primary objective of this study was to evaluate the practicality and adaptability of employing UCA-assisted US-guided PCN, and to determine if it could potentially substitute fluoroscopy in certain scenarios. Moreover, this is a single center study with relatively narrow scope of clinical symptoms. Only 13 patients were evaluated. Yet, over the past seven years, our conducted at an effectively substantiated the safety and dependability of this technology. Currently, we are conducting a multicenter trial to evaluate complications and ensure a longer follow-up period.

## Conclusion

While further assessment is required in a larger population and comparison with alternative methods, the combination of UCA and US-guided PCN shows promise in achieving urinary diversion and relieving clinical symptoms in non-hydronephrotic patients with urinary leakage. This technique has demonstrated a high success rate and therapeutic impact, with an acceptable incidence of complications. The favorable safety profile of UCA and the advantages offered by US, such as absence of radiation exposure, real-time performance, and bedside accessibility, make the amalgamation of UCA and US-guided PCN a potential novel diagnostic and therapeutic approach for urological applications.

## Data Availability

The datasets used and analyzed during the current study are available from the corresponding author on reasonable request.

## Abbreviations

US: Ultrasonography
PCN: Percutaneous nephrostomy
UCA: Ultrasound contrast agent
CT: Computed tomography
MBs: Micro bubbles
